# Outbreak investigations--a perspective.

**DOI:** 10.3201/eid0401.980104

**Published:** 1998

**Authors:** A L Reingold

**Affiliations:** School of Public Health, University of California,Berkeley 94720-7360, USA. reingold@uclink3.berkeley.edu

## Abstract

Outbreak investigations, an important and challenging component of epidemiology and public health, can help identify the source of ongoing outbreaks and prevent additional cases. Even when an outbreak is over, a thorough epidemiologic and environmental investigation often can increase our knowledge of a given disease and prevent future outbreaks. Finally, outbreak investigations provide epidemiologic training and foster cooperation between the clinical and public health communities.


### Outbreak Investigations—A Perspective

**Arthur L. Reingold**
University of California, Berkeley, California, USA

Outbreak investigations, an important and challenging component of epidemiology and public health, can help identify the source of ongoing outbreaks and prevent additional cases. Even when an outbreak is over, a thorough epidemiologic and environmental investigation often can increase our knowledge of a given disease and prevent future outbreaks. Finally, outbreak investigations provide epidemiologic training and foster cooperation between the clinical and public health communities.

Investigations of acute infectious disease outbreaks are very common, and the results of such investigations are often published; however, surprisingly little has been written about the actual procedures followed during such investigations (1,2). Most epidemiologists and public health officials learn the procedures by conducting investigations with the initial assistance of more experienced colleagues. This article outlines the general approach to conducting an outbreak investigation. The approach applies not only to infectious disease outbreaks but also to outbreaks due to noninfectious causes (e.g., toxic exposure).

### How Outbreaks Are Recognized

Possible outbreaks of disease come to the attention of public health officials in various ways. Often, an astute clinician, infection control nurse, or clinical laboratory worker first notices an unusual disease or an unusual number of cases of a disease and alerts public health officials. For example, staphylococcal toxic shock syndrome and eosinophilia myalgia syndrome were first noted by clinicians (3,4). Frequently, it is the patient (or someone close to the patient) who first suspects a problem, as is often the case in foodborne outbreaks after a shared meal and as was the case in the investigation of a cluster of cases of apparent juvenile rheumatoid arthritis near Lyme, Connecticut, which led to the discovery of Lyme disease (5). Review of routinely collected surveillance data can also detect outbreaks of known diseases, as in the case of hepatitis B infection among the patients of an oral surgeon in Connecticut and patients at a weight reduction clinic (6,7). The former outbreak was first suspected when routinely submitted communicable disease report forms for several patients from one small town indicated that all of the patients had recently had oral surgery. However, it is relatively uncommon for outbreaks to be detected in this way and even more uncommon for them to be detected in this way while they are still in progress. Finally, sometimes public health officials learn about outbreaks of disease from the local newspaper or television news.

Address for correspondence: Arthur L. Reingold, Division of Public Health Biology and Epidemiology, School of Public Health, University of California, Berkeley, 140 Warren Hall, Berkeley, CA 94720-7360, USA; fax: 510-643-5163; e-mail: reingold@uclink3.berkeley.edu.

### Reasons for Investigating Outbreaks

The most compelling reason to investigate a recognized outbreak of disease is that exposure to the source(s) of infection may be continuing; by identifying and eliminating the source of infection, we can prevent additional cases. For example, if cans of mushrooms containing botulinum toxin are still on store shelves or in homes or restaurants, their recall and destruction can prevent further cases of botulism.

However, even if an outbreak is essentially over by the time the epidemiologic investigation begins—that is, if no one is being further exposed to the source of infection—investigating the outbreak may still be indicated for many reasons. Foremost is that the results of the investigation may lead to recommendations or strategies for preventing similar future outbreaks. For example, a Legionnaires' disease outbreak investigation may produce recommendations for grocery store misting machine use that may prevent other outbreaks (8). Other reasons for investigating outbreaks are the opportunity to 1) describe new diseases and learn more about known diseases; 2) evaluate existing prevention strategies, e.g., vaccines; 3) teach (and learn) epidemiology; and 4) address public concern about the outbreak.

Once a decision is made to investigate an outbreak, three types of activities are generally involved—the epidemiologic investigation; the environmental investigation; and the interaction with the public, the press, and, in many instances, the legal system. While these activities often occur simultaneously throughout the investigation, it is conceptually easier to consider each of them separately.

### Epidemiologic Investigation

Outbreak investigations are, in theory, indistinguishable from other epidemiologic investigations; however, outbreak investigations encounter more constraints. 1) If the outbreak is ongoing at the time of the investigation, there is great urgency to find the source and prevent additional cases. 2) Because outbreak investigations frequently are public, there is substantial pressure to conclude them rapidly, particularly if the outbreak is ongoing. 3) In many outbreaks, the number of cases available for study is limited; therefore, the statistical power of the investigation is limited. 4) Early media reports concerning the outbreak may bias the responses of persons subsequently interviewed. 5) Because of legal liability and the financial interests of persons and institutions involved, there is pressure to conclude the investigation quickly, which may lead to hasty decisions regarding the source of the outbreak. 6) If detection of the outbreak is delayed, useful clinical and environmental samples may be very difficult or impossible to obtain.

Outbreak investigations have essential components as follows: 1) establish case definition(s); 2) confirm that cases are “real”; 3) establish the background rate of disease; 4) find cases, decide if there is an outbreak, define scope of the outbreak; 5) examine the descriptive epidemiologic features of the cases; 6) generate hypotheses; 7) test hypotheses; 8) collect and test environmental samples; 9) implement control measures; and 10) interact with the press, inform the public. While the first seven components are listed in logical order, in most outbreak investigations, many occur more or less simultaneously. The importance of these components may vary depending on the circumstances of a specific outbreak.

### Case Definition

In some outbreaks, formulating the case definition(s) and exclusion criteria is straightforward; for example, in an outbreak of gastroenteritis caused by Salmonella infection, a laboratory-confirmed case would be defined as a culture-confirmed infection with Salmonella or perhaps with Salmonella of the particular serotype causing the outbreak, while a clinical case definition might be new onset of diarrhea. In other outbreaks, the case definition and exclusion criteria are complex, particularly if the disease is new and the range of clinical manifestations is unknown (e.g., in a putative outbreak of chronic fatigue syndrome). In many outbreak investigations, multiple case definitions are used (e.g., laboratory-confirmed case vs. clinical case; definite vs. probable vs. possible case; outbreak-associated case vs. nonoutbreak-associated case, primary case vs. secondary case) and the resulting data are analyzed by using different case definitions. When the number of cases available for study is not a limiting factor and a case-control study is being used to examine risk factors for becoming a case, a strict case definition is often preferable to increase specificity and reduce misclassification of disease status (i.e., reduce the chance of including cases of unrelated illness or no illness as outbreak-related cases).

### Case Confirmation

In certain outbreaks, clinical findings in reported cases should be reviewed closely, either directly, by examining the patients, or indirectly, by detailed review of the medical records and discussion with the attending health-care provider(s), especially when a new disease appears to be emerging (e.g., in the early investigations of Legionnaires' disease, AIDS, eosinophilia myalgia syndrome, and hantavirus pulmonary syndrome) (4,9-11). Clinical findings should also be examined closely when some or all of the observed cases may be factitious, perhaps because of laboratory error (12); a discrepancy between the clinical and laboratory findings generally exists, which may be discernible only by a detailed review of the clinical findings.

### Establishing the Background Rate of Disease and Finding Cases

Once it is clear that a suspected outbreak is not the result of laboratory error, a set of activities should be undertaken to establish the background rate of the disease in the affected population and to find all the cases in a given population in a certain period. This set of activities should prove that the observed number of cases truly is in excess of the "usual" number (i.e., that an outbreak has occurred), define the scope of the outbreak geographically and temporally, find cases to describe the epidemiologic features of those affected and to include them in analytic epidemiologic studies (see below) or, most often, accomplish a combination of these goals.

When hundreds of acute onset diarrhea cases are suddenly seen daily in a single outpatient setting (10), an outbreak is clearly occurring. On the other hand, when too many hospitalized patients are dying unexpectedly of cardiac arrest (13) or the number of cases of listeriosis in a given county in recent months is moderately elevated, it may be necessary to establish the background rates in the population to determine whether an outbreak is occurring. In such situations, the period and geographic areas involved would provide the most useful baseline data, keeping in mind that the labor and time required to collect such information is often directly proportional to the length of the period and the size of the geographic area selected. Because disease incidence normally fluctuates by season, data from comparable seasons in earlier years should be included.

Establishing the background rate of a disease is generally more straightforward if confirmatory tests are available than if laboratory tests are unavailable or infrequently used. The rate of certain invasive bacterial infections (e.g., listeriosis and meningococcal infections) in a given area can be easily documented by reviewing the records of hospital clinical microbiology laboratories; however, cases for which specimens were not submitted to these laboratories for testing will go undetected. When a disease is less frequently laboratory-confirmed because health-care providers may not have considered the diagnosis or ordered the appropriate laboratory tests (e.g., for Legionnaires' disease), establishing the background rate of disease in a community or a hospital suspected of having an outbreak generally requires alternative case-finding strategies and is almost invariably more labor intensive. In an outbreak of a new disease, substantial effort is often necessary to determine whether or not cases of that disease had been occurring but had gone unrecognized.

Once data concerning the background rate of a disease (including case-finding for the current period) have been collected, it is generally possible to determine whether or not an outbreak is occurring or has occurred, although in some situations it may remain unclear whether or not the number of cases observed exceeds the background rate. In part, the problem may relate to how an outbreak is defined. To paraphrase a U.S. Supreme Court justice speaking about pornography, "I can't define an outbreak, but I know one when I see one." Thus, it may be difficult to detect and prove the existence of small outbreaks, but large ones are self-evident.

An outbreak can also be difficult to identify when during the period under study changes occur in the care-seeking behavior and access to care of patients; the level of suspicion, referral patterns, and test-ordering practices of health-care providers; the diagnostic tests and other procedures used by laboratories; and the prevalence of underlying immunosuppressive conditions or other host factors in the population. All these factors, which can affect the apparent incidence of a disease and produce artifactual changes perceived as increases (or decreases) in the actual incidence, need to be considered when interpreting the findings.

### Descriptive Epidemiology

By collecting patient data, the case-finding activities provide extremely important information concerning the descriptive epidemiologic features of the outbreak. By reviewing and plotting on an "epidemic curve" the times of onset of the cases and by examining the characteristics (e.g., age, sex, race/ethnicity, residence, occupation, recent travel, or attendance at events) of the ill persons, investigators can often generate hypotheses concerning the cause(s)/source(s) of the outbreak. While linking the sudden onset of gastroenteritis among scores of persons who attended a church supper to the single common meal they shared is generally not a challenge, an otherwise cryptic source can be at least hinted at by the descriptive epidemiologic features of the cases involved. For example, in a particularly perplexing outbreak of *Salmonella* Muenchen infections ultimately traced to contaminated marijuana, the age distribution of the affected persons and of their households was markedly different from that typically seen for salmonellosis (14). Or, similarly, in the outbreak of legionellosis due to contaminated misting machines in the produce section of a grocery store, before the link to this exposure was even suspected, it was noted that women constituted a substantially higher proportion of the cases usually seen with this disease (5). The shape of the epidemic curve can also be very instructive, suggesting a point-source epidemic, ongoing transmission, or a combination of the two.

### Generating a Hypothesis

The source(s) and route(s) of exposure must be determined to understand why an outbreak occurred, how to prevent similar outbreaks in the future, and, if the outbreak is ongoing, how to prevent others from being exposed to the source(s) of infection. In some outbreaks, the source and route are obvious to those involved in the outbreak and to the investigators. However, even when the source of exposure appears obvious at the outset, a modicum of skepticism should be retained because the obvious answer is not invariably correct. For example, in an outbreak of nosocomial legionellosis in Rhode Island, the results of an earlier investigation into a small number of hospital-acquired cases at the same hospital had demonstrated that *Legionella pneumophila* was in the hospital potable water supply, and a sudden increase in new cases was strongly believed to be related to the potable water (15). However, a detailed epidemiologic investigation implicated a new cooling tower at the hospital as the source of the second outbreak.

While the true source of exposure, or at least a relatively short list of possibilities, is apparent in many outbreaks, this is not the case in the more challenging outbreaks. In these instances, hypotheses concerning the source/route of exposure can be generated in a number of ways beyond a detailed review of the descriptive epidemiologic findings. A review of existing epidemiologic, microbiologic, and veterinary data is very useful for learning about known and suspected sources of previous outbreaks or sporadic cases of a given infection or disease, as well as the ecologic niche of an infectious agent. Thus, in an outbreak of invasive *Streptococcus zooepidemicus* infections in New Mexico due to consumption of soft cheese made from contaminated raw milk, the investigation focused on exposure to dairy products and animals because of previous microbiologic and veterinary studies (16).

A review of existing data generally only helps confirm what is already known about a particular disease and is far less helpful in identifying totally new and unsuspected sources or routes of infection (i.e., marijuana as a source of Salmonella). When neither review of the descriptive epidemiologic features of the cases nor review of existing scientific information yields the correct hypothesis, other methods can be used to generate hypotheses about what the patients have in common. Open-ended interviews of those infected (or their surrogates) are one such method in which investigators try to identify all possibly relevant exposures (e.g., a list of all foods consumed) during a given period. For example, in an investigation of *Yersinia enterocolitica* infections in young children in Belgium, open-ended interviews of the mothers of some of the ill children showed that many gave their children raw pork sausage as a weaning food, providing the first clue as to the source of these infections (17). Similarly, in two outbreaks of foodborne listeriosis, a variant of this process led to the identification of the source of the outbreak. In one of these outbreaks, a search of the refrigerator of one of the case-patients who, as a visitor to the area, had had very limited exposure to foods there, suggested cole slaw as a possible vehicle of infection (18). In the other outbreak, an initial case-control study found no differences between cases and controls regarding exposure to a number of specific food items but showed that case households were more likely than control households to buy their food at a particular foodstore chain. To generate a list of other possible food sources of infection, investigators shopped with persons who did the shopping for case households and compiled a list of foods purchased at that foodstore chain that had not been reported in the previous study. This approach implicated pasteurized milk from that chain as the source of the outbreak (19).

In some particularly perplexing outbreaks, bringing together a subset of the patients to discuss their experiences and exposures in a way that may reveal unidentified links can be useful.

### Testing the Hypothesis

Whether a hypothesis explaining the occurrence of an outbreak is easy or difficult to generate, an analytic epidemiologic study to test the proposed hypothesis should be considered. While in many instances a case-control study is used, other designs, including retrospective cohort and cross-sectional studies, can be equally or more appropriate. The goal of all these studies is to assess the relationship between a given exposure and the disease under study. Thus, each exposure of interest (e.g., each of the meals eaten together by passengers on a cruise ship and each of the foods and beverages served at those meals) constitutes a separate hypothesis to be tested in the analytic study. In outbreaks where generating the correct hypothesis is difficult, multiple analytic studies, with additional hypothesis-generating activities in between, are sometimes needed before the correct hypothesis is formed and tested (19).

In interpreting the results of such analytic studies, one must consider the possibility that "statistically significant" associations between one or more exposures and the disease may be chance findings, not indicative of a true relationship. By definition, any "statistically significant" association may have occurred by chance. (When the standard cut point of $p < 0.05$ is used, this occurs 5% of the time.) Because many analytic epidemiologic studies of outbreaks involve testing many hypotheses, the problem of "multiple comparisons" arises often.

While there are statistical methods for adjusting for multiple comparisons, when and even whether to use them is controversial. At a minimum, it is important to go beyond the statistical tests and examine the magnitude of the effect observed between exposure and disease (e.g., the odds ratio, relative risk) and the 95% confidence intervals, as well as biologic plausibility in deciding whether or not a given "statistically significant" relationship is likely to be biologically meaningful. Evidence of a dose-response effect between a given exposure and illness (i.e., the greater the exposure, the greater the risk for illness) makes a causal relationship between exposure and disease more likely. Whether the time interval between a given exposure and onset of illness is consistent with what is known about the incubation period of the disease under study must also be assessed. When illness is "statistically significantly" related to more than one exposure (e.g., to eating each of several foods at a common meal), it is important to determine whether multiple sources of infection (perhaps due to cross-contamination) are plausible and whether some of the noted associations are due to confounding (e.g., exposure to one potential source is linked to exposure to other sources) or to chance.

When trying to decide if a "statistically significant" exposure is the source of an outbreak, it is important to consider what proportion of the cases can be accounted for by that exposure. One or more of the patients may be classified as "nonexposed" for various reasons: incorrect information concerning exposure status (due to poor memory, language barriers); multiple sources of exposure or routes of transmission (perhaps due to cross-contamination); secondary person-to-person transmission that followed a common source exposure; or patients without the suspected exposure, representing background cases of the disease unrelated to the outbreak. The plausibility of each of these explanations varies by outbreak. While there is no cutoff point above or below which the proportion of exposed case-patients should fall before an exposure is thought to account for an outbreak, the lower this proportion, the less likely the exposure is, by itself, the source.

Other possibilities need to be considered when the analytic epidemiologic study finds no association between the hypothesized exposures and risk for disease. The most obvious possibility is that the real exposure was not among those examined, and additional hypotheses should be generated. However, other possibilities should also be considered, particularly when the setting of the outbreak makes this first explanation unlikely (e.g., when it is known that those involved in the outbreak shared only a single exposure or set of exposures, such as eating a single common meal). Two other explanations for failing to find a "statistically significant" link between one or more exposures and risk for illness also need to be considered—the number of persons available for study and the accuracy of the available information concerning the exposures. Thus, if the outbreak involves only a small number of cases (and non-ill persons), the statistical power of the analytic study to find a true difference in exposure between the ill and the non-ill (or a difference in the rate of disease among the exposed and the unexposed) is very limited. If the persons involved in the outbreak do not provide accurate information about their exposure to suspected sources or vehicles of infection because of lack of knowledge, poor memory, language difficulty, mental impairment, or other reasons, the resulting misclassification of exposure status also can prevent the epidemiologic study from implicating the source of infection. Studies have documented that even under ideal circumstances, memory concerning such exposures is faulty (20). However, given the usually enormous differences in rates of disease between those exposed and those not exposed to the source of the outbreak, even small studies or studies with substantial misclassification of exposure can still correctly identify the source.

### Environmental Investigation

Samples of foods and beverages served at a common meal believed to be the source of an outbreak of gastroenteritis or samples of the water or drift from a cooling tower believed to be the source of an outbreak of Legionnaires' disease can support epidemiologic findings. In the best scenario, the findings of the epidemiologic investigation would guide the collection and testing of environmental samples. However, environmental specimens often need to be obtained as soon as possible, either before they are no longer available, as in the case of residual food from a common meal, or before environmental interventions are implemented, as in the case of treating a cooling tower to eradicate Legionella. Because laboratory testing of environmental samples is often expensive and labor-intensive, it is sometimes reasonable to collect and store many samples but test only a limited number. Collaborating with a sanitarian, environmental engineer, or other professional during an environmental inspection or collection of specimens is always beneficial.

While finding or not finding the causative organism in environmental samples is often perceived by the public, the media, and the courts as powerful evidence implicating or exonerating an environmental source, either positive or negative findings can be misleading for several reasons. For example, finding Legionella in a hospital potable water system does not prove that the potable water (rather than a cooling tower or some other source) is responsible for an outbreak of Legionnaires' disease (21). Similarly, not finding the causative organism in an environmental sample does not conclusively rule out a source as the cause of the problem, in part because the samples obtained and tested may not represent the source (e.g., because of error in collecting the specimens, intervening changes in the environmental source) and in part because the samples may have been mishandled. Furthermore, in some outbreaks caused by well-characterized etiologic agents, laboratory methods of detecting the agent in environmental samples are insensitive, technically difficult, or not available, as in the case of recent outbreaks of *Cyclospora* infections associated with eating imported berries (22,23).

### Control Measures

Central to any outbreak investigation is the timely implementation of appropriate control measures to minimize further illness and death. At best, the implementation of control measures would be guided by the results of the epidemiologic investigation and possibly (when appropriate) the testing of environmental specimens. However, this approach may delay prevention of further exposure to a suspected source of the outbreak and is, therefore, unacceptable from a public health perspective. Because the recall of a food product, the closing of a restaurant, or similar interventions can have profound economic and legal implications for an institution, a manufacturer or owner, and the employees of the establishments involved, acting precipitously can also have substantial negative effects. The recent attribution of an outbreak of *Cyclospora* infections to strawberries from California demonstrates the economic impact that can result from releasing and acting on incorrect information (22,23). Thus, the timing and nature of control measures are difficult. Balancing the responsibility to prevent further disease with the need to protect the credibility and reputation of an institution is very challenging.

### Interactions with the Public and Press

While the public and the press are not aware of most outbreak investigations, media attention and public concern become part of some investigations. Throughout the course of an outbreak investigation, the need to share information with public officials, the press, the public, and the population affected by the outbreak must be assessed. While press, radio, and television reports can at times be inaccurate, overall the media can be a powerful means of sharing information about an investigation with the public and disseminating timely information about product recalls.

Dr. Reingold worked as an epidemiologist at the Centers for Disease Control and Prevention for 8 years before joining the faculty of the School of Public

Health at the University of California, Berkeley. He is currently professor of epidemiology and head of the Division of Public Health Biology and Epidemiology.

### References

1. Goodman RA, Buehler JW, Koplan JP. The epidemiologic field investigation: science and judgment in public health practice. Am J Epidemiol 1990;132:9-16.
2. MacKenzie WR, Goodman RA. The public health response to an outbreak. Current Issues in Public Health1996;2:1-4.
3. Chesney PJ, Chesney RW, Purdy W, Nelson D, McPherson T, Wand P, et al. Epidemiologic notes and reports: toxic-shock syndrome—United States. MMWR Morb Mortal Wkly Rep 1980;29:229-30.
4. Hertzman PA, Blevins WL, Mayer J, Greenfield B, Ting M, Gleich GJ, et al. Association of eosinophilia-myalgia syndrome with the ingestion of tryptophan. N Engl J Med 1980;322:871.
5. Steere AC, Malawista SE, Syndman DR, Shope RF, Andman WA, Ross MR, Steele FM. Lyme arthritis: an epidemic of oligoarticular arthritis in children and adults in three Connecticut communities. Arthritis Rheum 1977;20:7.
6. Reingold AL, Kane MA, Murphy BL, Checko P, Francis DP, Maynard JE. Transmission of Hepatitis B by an oral surgeon. J Infect Dis 1982;145:262.
7. Canter J, Mackey K, Good LS, Roberto RR, Chin J, Bond WW, et al. An outbreak of hepatitis B associated with jet injections in a weight reduction clinic. Arch Intern Med 1990;150:1923-7.
8. Mahoney FJ, Hoge CW, Farley TA, Barbaree JM, Breiman RF, Benson RF, McFarland LM. Communitywide outbreak of Legionnaires' disease associated with a grocery store mist machine. J Infect Dis 1992;165:736.
9. Fraser DW, Tsai TR, Orenstein W, Parkin WE, Beecham HJ, Sharrar RG, et al. Legionnaires' disease: description of an epidemic of pneumonia. N Engl J Med 1977;297:1189-97.
10. Kriedman-Kien A, Laubenstein L, Marmor M, Hymes K, Green J, Ragaz A, et al. Kaposi's sarcoma and *Pneumocystis* pneumonia among homosexual men—New York City and California. MMWR Morb Mortal Wkly Rep 1981;30:305-8.
11. Koster F, Levy H, Mertz G, Young S, Foucar K, McLaughlin J, et al. Outbreak of acute illness—southwestern United States. MMWR Morb Mortal Wkly Rep 1993;42:421-4.
12. Weinstein RA, Bauer FW, Hoffman RD, Tyler PG, Anderson RL, Stamm WE. Factitious meningitis: diagnostic error due to nonviable bacteria in commercial lumbar puncture trays. JAMA 1975;233:878.
13. Buehler JW, Smith LF, Wallace EM, Heath CW, Rusiak R, Herndon JL. Unexplained deaths in a children's hospital: an epidemiologic assessment. N Engl J Med 1985;313:211.
14. Taylor DN, Wachsmuth IK, Shangkuan Y-H, Schmidt EV, Barrett TJ, Schrader JS, et al. Salmonellosis associated with marijuana: a multistate outbreak traced by plasmid fingerprinting. N Engl J Med 1982;306:1249.
15. Garbe PL, Davis BJ, Weisfeld JS, Markowitz L, Miner P, Garrity F, et al. Nosocomial Legionnaires' disease: epidemiologic demonstration of cooling towers as a source. JAMA 1985;254:521.
16. Espinosa FH, Ryan WM, Vigil PL, Gregory DF, Hilley RB, Romig DA, et al. Group C streptococcal infections associated with eating homemade cheese: New Mexico. MMWR Morb Mortal Wkly Rep 1983;32:514.
17. Tauxe RV, Walters G, Goossen V, VanNoyer R, Vandepitte J, Martin SM, et al. *Yersinia enterocolitica* infections and pork: the missing link. Lancet 1987;5:1129.
18. Schlech WF, Lavigne PM, Bortolussi RA, Allen AC, Haldane EV, Wort AJ, et al. Epidemic listeriosis: evidence for transmission by food. N Engl J Med 1983;308:203.
19. Fleming DW, Cochi SL, MacDonald KL, Brondum J, Hayes PS, Plikaytis BD, et al. Pasteurized milk as a vehicle of infection in an outbreak of listeriosis. N Engl J Med 1985;312:404.
20. Decker MD, Booth AL, Dewey MJ, Fricker RS, Hutcheson RH, Schaffner W. Validity of food consumption histories in a foodborne outbreak investigation. Am J Epidemiol 1986;124:859.
21. Hayes EB, Matte TD, O'Brien TR, McKinley TW, Logsdon GS, Rose JB, et al. Large community outbreak of cryptosporidiosis due to contamination of a filtered public water supply. N Engl J Med 1989;390:1372.
22. Chambers J, Somerfieldt S, Mackey L, Nichols S, Ball R, Roberts D, et al. Outbreaks of *Cyclospora cayetanensis* infection—United States, 1996. MMWR Morb Mortal Wkly Rep 1996;45:549-51.
23. Hofman J, Liu Z, Genese C, Wolf G, Manley W, Pilot K, et al. Update: outbreaks of *Cyclospora cayetanensis* infection—United States and Canada, 1996. MMWR Morb Mortal Wkly Rep 1996;45:611-2.
